# Malaria and Helminth Coinfection among Children at the Douala Gyneco-Obstetric and Pediatric Hospital

**DOI:** 10.1155/2021/3702693

**Published:** 2021-07-01

**Authors:** Ashungafack Flavio, Yamssi Cedric, Noumedem Anangmo Christelle Nadia, Vincent Khan Payne

**Affiliations:** ^1^Department of Animal Biology, Faculty of Science, University of Dschang, P. O. Box 067, Dschang, Cameroon; ^2^Department of Biomedical Sciences, Faculty of Health Sciences, University of Bamenda, P. O. Box 39, Bambili, Cameroon; ^3^Department of Microbiology, Hematology and Immunology Faculty of Medicine and Pharmaceutical Sciences, University of Dschang, P. O. Box 96, Dschang, Cameroon

## Abstract

**Background:**

Malaria and helminth infections are major public health issues in sub-Saharan Africa including Cameroon. This study was aimed at determining the prevalence and risk factors associated with malaria and helminth coinfection among children in the Douala Gyneco-Obstetric and Pediatric Hospital (HGOPED) in Douala, southwestern Cameroon. *Material and Methods*. The study was a hospital-based cross-sectional study that took place from January to July 2020 where 203 children were involved. Blood samples were collected from the children and thick blood smears were prepared and examined microscopically for malaria parasites. Stool samples were also collected and examined through the Kato-Katz technique for the identification of helminth eggs. Demographic and socioeconomic data and information of participant's knowledge on the transmission of malaria and helminth infections were collected with the use of a well-structured questionnaire.

**Results:**

The overall prevalence of *P. falciparum* infection was 28.8%, while the overall prevalence of helminth was 9.36%. The only species of helminth identified were *Ascaris lumbricoides* and *Trichuris trichiura* with a prevalence of 4.26% and 2.95%, respectively, and mixed infection of both *A. lumbricoides* and *T. trichiura* with a prevalence of 1.47%. Coinfection of malaria and helminth was observed with a prevalence of 6.90%. Associations of malaria-helminth coinfection with age groups, parent's educational level, type of latrine, and source of water factors were not statistically significant (*p* > 0.05), while the prevalence of the coinfection with respect to parent's occupation, presence of stagnant water around homes, washing of hands after using the toilet, and washing of fruits before eating was statistically significant (*p* < 0.05).

**Conclusion:**

The findings suggest that helminths and malaria infections tend to occur in children. Not washing hands after using the toilet, not washing fruits before eating, the presence of stagnant water around homes, and parents' occupation were found to be strongly associated with coinfection. Health education on the importance of better sewage disposal, draining of stagnant water around homes, and other sanitary practices is recommended.

## 1. Introduction

It is estimated that over a third of the world's population, mainly living in the tropics and subtropics, are infected by at least one intestinal helminth (worm) or a protozoan parasite species [1, 2] which, according to Degarege et al. [3], may adversely impact the outcome of the disease they cause.

Malaria is a febrile illness caused by an infectious parasitic protozoan of the genus *Plasmodium* through the bite of an infected female *Anopheles* mosquito [4]. Though it is caused by several *Plasmodium* protozoan parasites, *Plasmodium falciparum* is the most dangerous. Malaria is widespread in tropical and subtropical parts of America, Asia, and Africa. Each year, there are more than 250 million cases of malaria, killing between 1 and 3 billion people, the majority of whom are young children in sub-Saharan Africa [2]. Of all human diseases caused by protozoan parasites, malaria has the greatest burden and is responsible for the highest number of deaths among young children in sub-Saharan Africa, accounting for 90% of all global cases [5].

In addition to malaria, other parasitic diseases are common among the world's poorest people. According to Hotez and David [5], there are about 13 parasitic infections that are responsible for significant morbidity but have not gotten the attention they deserve; thus, they are now known as “Neglected Tropical Diseases” (NTDs). Three of these are soil-transmitted helminthic infections (STH) (*Ascaris lumbricoides*), whipworm (*Trichuris trichiura*), and hookworm (*Ancylostoma duodenale* and *Necator americanus*). They are so-called soil-transmitted helminths because their infection on man is transmitted via contaminated soil by parasitic invasive eggs or larvae.

In Cameroon, both malaria and helminth infections coexist and are ranked among the major causes of parasitic mortality and morbidity with *Plasmodium falciparum* being the most prevalent and virulent species of malaria parasite [6–8]. The prevalent and ubiquitous geohelminths *A. lumbricoides, T. trichiura, A. duodenale,* and *Strongyloides stercoralis* are the major helminth species [9–11]. This frustrates the health achievements in the millennium development goals and impedes public health outcomes [12].

This study aims to determine the prevalence and risk factors associated with malaria and helminth coinfection among children in the Douala Gyneco-Obstetric and Pediatric Hospital to contribute to the existing data on coinfection of malaria and helminth in Cameroon, making recommendations for further regional and nationwide control programs.

## 2. Materials and Methods

### 2.1. Study Site

The study was conducted in the Douala Gyneco-Obstetric and Pediatric Hospital (HGOPED). Douala, which is the largest city of Cameroon, is found in the littoral region of the country with around 2.5 million inhabitants. It is located at latitude 4°3'41.5296”N and longitude 9°47'9.8592”E within the Congo-Guinean phytogeographic zone near the Atlantic coast and lies about one meter above sea level.

### 2.2. Study Design and Sample Size

This study was a hospital-based cross-sectional study. The study population was made up of children (1 to 16 years old) of both sexes (male and female) present at the Douala Gyneco-Obstetric and Pediatric Hospital whose written and signed parental/guardian consent was obtained for participation in the study. The sample size was be determined using Lorenze's formula:

(1) $$\begin{array}{c} n=\frac{z^{2}pq}{d^{2}}, \end{array}$$

where *n* = required sample size, *Z* = confidence interval at 95% (standard value = 1.96), *P* = estimated prevalence, *q* (1−*p*) = expected nonprevalence, and *d* = margin of error at 5% (0.05) or maximum tolerable error.

The estimated prevalence of 22.1% (0.0221) was used:

(2) $$\begin{array}{c} n=\frac{{\left(1,96\right)}^{2}\left(0,0221\right)\left(0,929\right)}{{\left(0,05\right)}^{2}}=264.5. \end{array}$$

It is approximately 265 subjects.

#### 2.2.1. Inclusion Criteria

All children from 1 to 16 whose parents/guardians accepted to give his/her consent were included.

#### 2.2.2. Exclusion Criteria

Children whose parents/guardians refused to give his/her consent were immediately excluded.

### 2.3. Administration of Questionnaire

In order to collect primary data, all consented participants assisted by their parents/guardians were given a well-structured questionnaire designed to collect personal and sociodemographic information such as age and sex of the pupil, characteristics of their homes, and their knowledge on the transmission of malaria and soil-transmitted helminth infections.

### 2.4. Collection of Stool Samples

Well labelled sterile containers with individual identification number were distributed to all participants who were instructed on how to do a proper fresh fecal sample collection. The stool samples collected were taken to the laboratory and examined immediately.

### 2.5. Laboratory Analysis of Stool and Blood Samples Collection

Parasitological examination of stool sample was done using the Kato-Katz technique [12]. After sterilizing the fingertip, a drop of blood was collected by finger prick and thick blood smears were prepared for malaria parasite identification and examined under the microscope at a 10x objective.

### 2.6. Data Analysis

Data collected and stored in a computer using Microsoft Excel 2013 was transferred to XLSTAT. Data obtained was analyzed using descriptive statistics where variables were summarized using frequencies and percentages. Chi square test was used to test for the association between malaria and helminth coinfection and its related risk factors and Pearson correlation was used to test the strength of the association between variables. The statistical significance threshold was set at *p* < 0.05.

## 3. Results

Table 1 shows the prevalence of malaria and helminth infection. It follows from the analysis of this table that 57 (28.08%) of the patients were positive for malaria parasite, 24 (42.10%) males and 33 (57.89%) females. It also shows that 19 (9.36%) of the participants had soil-transmitted helminth infection. Of these 19, 11 (4.95%) had *A. lumbricoides* infections, 6 (2.95%) had *T. trichiura* infection, and 3 (1.47%) had both *A. lumbricoides* and *T. trichiura*.

Table 2 shows the prevalence of malaria and helminth coinfection. It appears from this table that, out of 203 participants, 14 (6.90%) had malaria and soil-transmitted helminth coinfection, 5 (2.46%) had soil-transmitted helminth infection, and 43 (21.187%) had malaria infection.

From the *p* values and Pearson correlation values presented on Table 3, it appears that washing hands after using the toilet was significantly associated with malaria-helminth coinfection (*p*=0.001). It also reveals that washing fruits before eating and patients' behavior of “sometimes” washing their fruits before eating were significantly associated with malaria-helminth coinfection (*p*=0.001); equally the presence of stagnant water and unemptied bottles around patient were significantly associated with coinfection (*p*=0.001). Parents' economic status of having “no job” was strongly associated with coinfection (*p*=0.043).

## 4. Discussion

This study shows an overall malaria prevalence of 28.8% which is slightly similar to 33% observed by Laurantine et al. [12] in Tombel health district. This similarity in the observed prevalence may be due to similarity in climatic conditions responsible for the development of malaria vector in these two regions. This observed prevalence however differs from 45.47% malaria prevalence obtained by Leopold et al. [13] from their study on the epidemiology of malaria among school children in Douala. This may be explained by the fact that though their study population was on children as in this study, it only included children of 3 to 16 years; meanwhile this study includes children of 1 to 16 years. Also, their study design was school-based while our study was hospital-based.

The overall prevalence of STH registered in this study was 8.87% which is lower compared to that observed in a similar study conducted by Zeukeng et al. [11] in the Mfou health district of the central region. This may be explained by the fact that Mfou district is in a rural area while HGOPED where this study was conducted is in an urban center with more access to pipe-borne water, hence lower chances of transmission by water contamination. Again, the study in Mfou district was conducted only in rural communities where farming and animal rearing are often the major occupations in rural areas; this increases the chances of soil-transmitted helminth. Also, no cases of hookworm infection were identified in this study; this confirms the result of studies conducted by Makoge et al. [14] in Mbonge and Tchinda et al. [7] in Mfou health district but disagrees with the study of Pone et al. [15] in Dschang and Degarege et al. [3]. The absence of hookworm may be associated with the type of soil in the area which may not be favorable for larval development. It may also be due to the time between stool processing and microscopy since hookworms start to degenerate after about 45 minutes of Kato-Katz preparation.

The overall prevalence of malaria-helminth coinfection obtained from this study was 6.8% which was similar to 7.1% prevalence obtained by Laurantine et al. [12] on a similar study in Tombel health district. This similarity in prevalence may be due to the similarity in the climatic conditions of the study area. It however differs from the prevalence of 22.1% observed by Zeukeng et al. [11] in Mfou health district of the central region. This may likely be because the study of Zeukeng was centered on all age groups, in the rural areas and with a large sample size; meanwhile, this study was only centered on children of age 1–16 years in HGOPED which is in an urban area and only 203 children were sampled.

There was a higher prevalence (51.14%) of malaria-helminth coinfection in children less than 5 years of age than in other age groups. This differs from the report of Zeukeng et al. [11] in Mfou health district where they reported a high prevalence of coinfection of malaria and helminth only in children between 5 and 11 years. But the high prevalence of coinfection among children less than 5 is not surprising because children at this age have not yet acquired strong immunity against malaria and they still have the habit of crawling and eating the soil which may be contaminated with helminth eggs.

Based on the parent's occupation, a high prevalence of coinfection of 8 (87.14) was recorded in children whose parents were self-employed (*p*=0.036), followed by no job and casual workers compared with no case of coinfection observed in children of civil servants. This may be explained by the fact that parents with no job, casual workers, and self-employed ones have low household income leading to poor feeding habits and consequently coinfection. This observation agrees with a previous study conducted by Lanne [16] in Sierra Leon where he reported that low family income of less than 50 USD was statistically more associated with malaria-helminth coinfection in children.

It was also observed that poor sanitary practices such as not washing hands after using the toilet, not washing fruits before eating (*p*=0.001), and the presence of stagnant water pools around homes were strongly associated with malaria-helminth coinfection. This agrees with the previous studies conducted by Nkuo-Akenji et al. [6] in the Southwest Region of Cameroon and Messina et al. [17] in Congo where they reported that environmental factors like stagnant water, bushes around homes, unhygienic conditions, and high temperatures favor the growth and transmission of both malaria and soil-transmitted helminth. This is not surprising because unwashed hands and fruits contain dust contaminated with eggs of helminth and meanwhile stagnant water pools serve as breeding grounds for *Anopheles* mosquitos, hence making coinfection inevitable.

## 5. Conclusion

The study reveals that there is coinfection of malaria and helminth among children in the Douala Gyneco-Obstetric and Pediatric Hospital. Poor hygiene practices such as not or “sometimes” washing hands after using the toilet, not washing fruits before eating, poverty resulting from parents having no job, and the presence of stagnant water pools around homes are significantly associated with malaria-helminth coinfection among children in the Douala Gyneco-Obstetric and Pediatric Hospital.

## Figures and Tables

**Table 1:** Prevalence of malaria and helminth infection and parasitic associations

Variable	Frequency (%)
**Malaria status**	
** Positive (n** **=** **57)**	**57 (28.08)**
** **Male	24 (42.10)
** **Female	33 (57.89)
** Negative (146)**	**146 (71.93)**
** **Male	74 (50.68)
** **Female	72 (49.32)

**Helminth infection (n** **=** **19)**	**19 (9.36)**
*Ascaris lumbricoides*	10 (4.26)
*Trichuris trichiura*	6 (2.95)
*Ascaris lumbricoides/Trichuris trichiura*	3 (1.47)

**Table 2:** Prevalence of malaria-helminth coinfection.

	Positive	Negative	Coinfection
Malaria	43 (28.08)	146 (71.92%)	14 (6.90%)
Helminth	5 (2.46%)	184 (90.64%)	

Risk factors associated with malaria-helminth coinfection.

**Table 3:** Risk factors associated with malaria-helminth coinfection.

Variable	Coinfection		Pearson correlation	*p* value
	Positive (*n* = 14)	Negative (*n* = 189)		
*Sex*				
Male	6 (42.56%)	90 (47.62%)	0.048	0.498
Female	8 (57.14%)	99 (52.88%)	0.048	0.494

*Age group*				
<5	8 (51.14%)	76 (40.21%)	0.087	0.51
5to 9	2 (14.28%)	90 (47.61%)	0.074	0.387
10 to 16	4 (28.57%)	23 (11.11%)	0.021	1.00

*Parent/guardian educational level*			**0.085**	**0.225**
Non	0%	20 (10.58%)	0.090	0.263
Primary	3 (21.43%)	51 (26.98%)	0.032	0.665
Secondary	8 (51.14%)	83 (43.92%)	0.067	0.855
Tertiary	3 (21.43%)	3 (21.43%)	0.019	1.00

*Parent occupation*			**0.147**	**0.036**
Casual	3 (21.43%)	38 (20.11%)	0.008	0.306
Civil servant	0 (0%)	20 (10.58%)	0.090	0.055
Self-employed	8 (57.14%)	75 (39.68%)	0.0150	1.00
No job	3 (21.43%)	56 (29.63%)	0.095	0.043

*Habitat type*				
Cemented	198 (97.33%)	185 (97.88%)	0.082	0.80
Plank	4 (1.97%)	3 (1.58%)	0.101	0.38
Others	1 (0.493%)	1 (0.53%)	0.019	1.00

*Water source*			**0.007**	**0.916**
Borehole	9 (64.29%)	114 (76.19%)	0.021	0.773
Pipe-borne	5 (35.71%)	54 (28.57%)	0.189	0.107
Other sources	0	21 (11.11%)	0.092	1.00

*Toilet facility*			**0.049**	**0.483**
Water closet	9 (64.29%)	137 (72.49%)	0.047	0.48
Pit latrine	5 (35.71%)	51 (26.98%)	0.049	0.48

*Washing hands after using the toilet*			**0.297**	**0.0001**
Yes	3 (21.43%)	48 (25.40%)	0.297	
No	0	0	--	
Sometimes	11 (78.57%)	141 (74.60%)	0.297	

*Washing of fruits before eating*			**0.274**	**0.0001**
Yes	4 (28.57%)	144 (76.19%)	0.019	0.000
No	0	0	0.276	0.913
Sometimes	10 (71.43%)	44 (81.48%)	0.271	0.000

*Stagnant water pools around homes*				**0.0001**
Yes	12 (85.71%)	122 (64.55%)		
No	2 (14.29%)	67 (34.44%)	0.261	

## Data Availability

Data and material are available upon request to the corresponding author.
